# On the proposed role of metacognition in environment learning: recommendations for research

**DOI:** 10.1186/s41235-022-00454-x

**Published:** 2022-12-27

**Authors:** Lauren A. Mason, Ayanna K. Thomas, Holly A. Taylor

**Affiliations:** 1 Department of Psychology, Tufts University, Medford, MA USA; 2 Center for Applied Brain and Cognitive Sciences, Tufts University, Medford, MA USA

**Keywords:** Environment learning, Metacognitive monitoring, Metacognitive control

## Abstract

Metacognition plays a role in environment learning (EL). When navigating, we monitor environment information to judge our likelihood to remember our way, and we engage in control by using tools to prevent getting lost. Yet, the relationship between metacognition and EL is understudied. In this paper, we examine the possibility of leveraging metacognition to support EL. However, traditional metacognitive theories and methodologies were not developed with EL in mind. Here, we use traditional metacognitive theories and approaches as a foundation for a new examination of metacognition in EL. We highlight three critical considerations about EL. Namely: (1) EL is a complex process that unfolds sequentially and is thereby enriched with multiple different types of cues, (2) EL is inherently driven by a series of ecologically relevant motivations and constraints, and (3) monitoring and control interact to support EL. In doing so, we describe how task demands and learning motivations inherent to EL should shape how metacognition is explored. With these considerations, we provide three methodological recommendations for investigating metacognition during EL. Specifically, researchers should: (1) instantiate EL goals to impact learning, metacognition, and retrieval processes, (2) prompt learners to make frequent metacognitive judgments and consider metacognitive accuracy as a primary performance metric, and (3) incorporate insights from both transfer appropriate processing and monitoring hypotheses when designing EL assessments. In summary, to effectively investigate how metacognition impacts EL, both ecological and methodological considerations need to be weighed.

## Introduction

Imagine a student is studying for an upcoming French vocabulary examination. They review pairs of translated words, such as *bike*–*vélo*. When looking at *bike*, they consider the likelihood of remembering *vélo* on the examination. This exemplifies a metacognitive judgment known as a judgment of learning (JOL). Metacognition consists of monitoring and control (Nelson & Narens, 1990, 1994). In the present review, we describe monitoring as one’s awareness of their own cognitive ability and learning success, while control consists of actions taken to modify the cognitive state and learning process in some way. For example, if the student thinks themself unlikely to remember that *vélo* means *bike* (monitoring), they might choose to restudy (control) this vocabulary pair.

This scenario is a classic account of how metacognition plays a regulatory role during word pair learning. Analogous laboratory-based studies inform how metacognition impacts verbal learning, potentially by influencing people’s learning goals (Janes et al., 2018) and how they attend to (Mitchum et al., 2016) or encode information (Double et al., 2018; Myers et al., 2020). Findings also suggest metacognition contributes to accurate mental model development during multimedia learning (Azevedo & Hadwin, 2005; Cuevas et al., 2002, 2004; Schwartz et al., 2004; Scott & Schwartz, 2007). Yet, the beneficial role of metacognition for complex environment learning (EL) is only of recent interest.

### Scope of the present review

In the present review, we provide support for the notion that metacognition may aid EL, with the critical caveat that to promote such gains, both ecological and methodological considerations must be weighed. To understand how metacognitive processes can scaffold EL, we first highlight important EL characteristics. With these considerations, we explain how these characteristics should guide theoretical and methodological approaches to understanding metacognition’s role in EL.

Only a handful of studies have explored metacognition in EL (Hegarty et al., 2012; Schwartz et al., 2004; Stevens & Carlson, 2016, 2019; Tenbrink & Salwiczek, 2016). As such, we are unaware of any review papers that describe the potential role of metacognition during EL or what to consider when conducting such research. With this review, we aim to provide a roadmap for future researchers interested in systematically investigating metacognition’s role in EL. We pull from several research domains, including verbal learning, multimedia learning, and spatial learning, to broadly cover the theoretical perspectives relevant to this novel research area. These are relevant areas to draw upon for two reasons. First, EL encompasses aspects of spatial and verbal learning. Second, environments can be learned from several modalities, including real-world navigation, VR navigation, multimedia map displays, and by receiving verbal directions from others.

We largely base our assumptions about metacognition’s beneficial role during EL on findings from the verbal learning literature. However, we acknowledge that environments are fundamentally different from word pairs. Namely, environments are complex, a notion reinforced throughout this review. Therefore, it is likely that metacognitive theories derived from verbal learning experiments will require rethinking in this EL context. Further research is needed to explicitly examine the extent to which this is the case. In the present review, we outline considerations one should have when examining metacognition and EL. We specifically focus on EL encoding, or situations where people learn a new environment. Then, we provide practical recommendations to better understand metacognitive processes in EL.

### Considering space and time as metacognitive cues in EL

Imagine an undergraduate steps foot on their college campus for the first time. This student will eventually know their campus layout, but first they must begin to actively explore and learn it. They travel from their dorm to the campus bookstore to buy textbooks. Along the way, they observe various landmarks, including the post office and psychology building to their left, and the library to their right. While walking, they note that the setting sun designates the western side of campus, where the bookstore is located. As they approach their destination, they reflect on whether they remember their way back to the dorm (monitoring) so that they can join their roommates for dinner. After purchasing their books, they decide they should review a map on their phone (control) to help quickly find their way back.

As garnered from this scenario, EL is an elaborate task that requires integrating information over time and space. Namely: (1) EL is a complex process that unfolds sequentially and is thereby enriched with multiple different types of cues, (2) EL is inherently driven by a series of ecologically relevant motivations and constraints, and (3) monitoring and control interact to support EL. Finally, task demands and learning motivations shape how metacognitive processes operate in EL.

#### External environment cues: global and local

Based on prior research, external environmental cues (allothetic cues) refer to inputs from the external world, including visual and auditory cues (Chen et al., 2017). These environmental cues can be subdivided based on whether they provide direct or indirect location information. In the above scenario, the buildings the student observes serve as allothetic cues that provide direct information about their location. We will refer to direct environment cues that are perceivable from a single vantage point as *local cues* (Meilinger et al., 2014). In contrast, the sun’s location in the sky provides indirect information that can be used to estimate position. The student monitors the sun to estimate cardinal directions and orient relative to their destination. If they are still uncertain about their position, they might metacognitively control learning by referring to a map for additional cardinal direction information. We refer to indirect environment cues that are perceivable from many vantage points as *global cues.*

#### Internal spatial cues

Another cue category is internal spatial cues, or cues that use the self (i.e., the navigator) as the reference point. Prior spatial research describes internal spatial cues as bodily information generated through self-movement (Chen et al., 2017), such as when the student notes the post office and psychology building locations relative to themself (e.g., on the left). Internal spatial cues provide information on our position in space, which can be used for both metacognitive monitoring (Stevens & Carlson, 2016) and control. We will refer to *internal spatial cues* to describe environment spatial features in reference to a navigator.

#### Mnemonic cues

The final cue category involves mnemonic cues. In the metacognitive literature, mnemonic cues refer to the learner’s subjective experience, including how easily information is processed or retrieved (Koriat, 1997) or uncertainty about one’s location (Keller et al., 2020). The student feeling uncertain when preparing to navigate back to the dorm exemplifies a mnemonic cue. Both external and internal factors can impact mnemonic cues. In terms of external uncertainty triggers, a distinctive sportscar (an unfixed landmark) the student remembers has likely moved when they re-navigate the route. With respect to internal factors, confidence in one’s wayfinding ability affects behavior. Prior experience with similar environments might also play a role in subjective wayfinding confidence. Scenes provide “semantic guidance,” allowing learners to draw on their rich knowledge base of scene priors (Võ & Wolfe, 2013). One might conjecture that prior knowledge about the landmarks that typically exist on a school campus could contribute to the student’s confidence that they will find a bookstore.

This sense of confidence, or subjective evaluation, is similarly described in both spatial and multimedia research (Chen et al., 2017; Cuevas et al., 2002). Subjective evaluation impacts participant responses and the value attributed to other environment cues (Chen et al., 2017). The student monitors this uncertainty and, if it is high, exhibits metacognitive control by reviewing a map (Dai et al., 2018). In other words, mnemonic cues also play a role in how metacognitive monitoring and control interact to support EL. We will refer to *mnemonic cues* when discussing the learner’s subjective experiences.

#### Cue frameworks

There are similarities between the cue framework categorized here and the cue-utilization framework proposed by Koriat (1997). Namely, three different cue types inform metacognitive judgments, and these cues may influence one another. There are also notable differences. In the cue-utilization framework, extrinsic cues involve learning conditions or cognitive operations applied by the learner, such as the number of items studied or the learner’s level of processing. In contrast, *external cues* in the present framework encompass inputs from the external world that provide direct (local) or indirect (global) spatial position information. Next, in Koriat’s framework, intrinsic cues involve item characteristics that communicate ease or difficulty of learning. In the current framework, *internal cues* relate environment features to the self (Levinson, 1996).

**Fig. 1:**
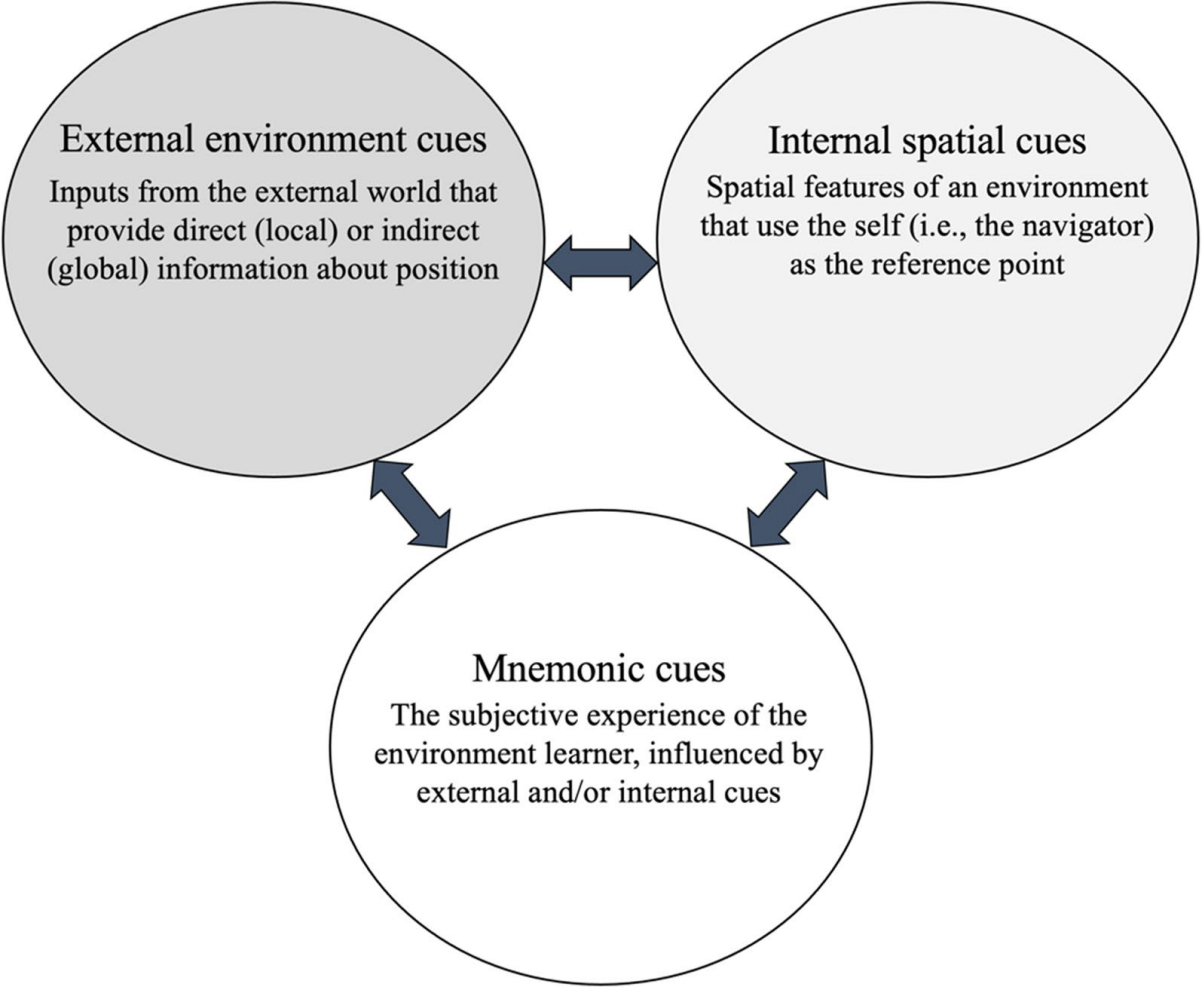
Cue categories and interactions. Monitoring these cues informs how learners metacognitively control the learning process

Finally, Koriat’s and the current framework overlap considerably with respect to mnemonic cues. An important distinction here is that *mnemonic cues* encompass the learner’s entire subjective experience, including that caused by theories about one’s own EL abilities. An example of an EL theory is, “I am bad with direction.” As determined by Koriat, mnemonic cues are sensitive to both external and internal factors. Figure 1 adds to this idea by introducing bidirectional relationships between all cue types. While mnemonic cues are sensitive to external environment cues and internal spatial cues, they can also lead learners to seek out additional information from these other cue types (Keller et al., 2020). This bidirectional relationship is also represented between internal and external cues because a navigator experiences inputs from the world (external environment cues) in reference to themself (internal spatial cues). Monitoring each of these cues informs the way learners control the learning process.

#### Cue combination

In summary, EL involves many cues, including external environment cues, internal spatial cues, and mnemonic cues. These cues combine to inform EL, but open questions remain as to how and when they combine (Chen et al., 2017; McNamara & Chen, 2022; Newman & McNamara, 2021; Ratliff & Newcombe, 2008; Selmeczy & Dobbins, 2017, 2017; Sjolund et al., 2018; Sun et al., 2022). Individuals can differentially weight EL cues based on a cue’s relative reliability and/or on subjective evaluation of performance with a given cue (Chen et al., 2017). Environment cues can also change over time (such as when new buildings are added), which impacts the cue’s utility to aid learning and memory. Furthermore, cue types and functions vary during EL. Specifically, external environment cues, internal spatial cues, and mnemonic cues interact to provide information about the external world, spatial features as they relate to the navigator, and the navigator’s subjective experience, respectively.

Further, little is known about which cues people refer to or combine to formulate metacognitive monitoring judgments during EL. While recent research reviews how navigators combine spatial cues to estimate target or goal locations (McNamara & Chen, 2022), the variety of cues people reference, and the cognitive process of combining such cues to estimate memorability, may fundamentally differ from estimating location alone. Although McNamara and Chen (2022) address how Bayesian decision theory can explain cue and location estimate biases, to our knowledge no reviews describe how people combine cues to make metacognitive judgments during EL. Cue combination processes have implications for metacognitive judgment formation and subsequent control processes in environment learners.

How people combine cues during EL likely depends on multiple factors, including how environment information is acquired (navigation, map, verbal directions), presence of distinctive landmarks, and individual differences. Certain cues may be more useful in some contexts than others. For example, internal spatial cues are less available when a friend is explaining their favorite coffee shop’s location over the phone or when you are looking for the coffee shop on a map than when navigating. However, external environment cues, such as distinctive landmarks near the coffee shop, may be useful in most contexts (Kelly et al., 2008; Torok et al., 2014).

To control for the influence of environmental cues on navigation, Tenbrink and Salwiczek (2016) had participants navigate and make direction judgments in an extremely simplistic space void of the complexity encountered in real environments. Even in the absence of environmental cues, the cognitive challenges induced by the direction judgment were profound enough to cause participants to engage in metacognitive strategies, such as employing conscious cognitive shortcuts *(the tunnel went left, therefore the entrance must be to the right)* and modifying their procedures to mitigate confusion. It is therefore reasonable to assume that in the presence of real-world environment cues, metacognitive processes are similarly engaged. Further, effectively monitoring mnemonic cues, such as uncertainty, may promote metacognitive control processes (Shields et al., 1997), such as controlling learning (seeking more locational details) to avoid negative outcomes. Finally, learner characteristics may influence variation in cue utilization. For instance, some individuals prefer local environment cues to guide navigation (I pass the post office, then turn right), whereas others refer primarily to global cues to orient themself (I go west until I reach the bookstore) (Boone et al., 2019; Brunyé et al., 2009, 2012; Pazzaglia & De Beni, 2001).

### Ecologically relevant motivations and constraints drive EL

EL is inherently driven by ecologically relevant motivations and constraints. Here, we use the term *motivations* to refer to goal states, while we use *constraints* to encompass limiting factors including *environment constraints, time constraints,* and *cognitive constraints.* These constraints subsequently shape possible methodological approaches in metacognition and EL research.

In the scenario above, the student has a variety of motivations while navigating (purchasing textbooks, meeting roommates at the dorm in time for dinner, etc.). Metacognitive processing may come into play to achieve these goals. For example, several motivations are active when the student assesses their likelihood to make it back to the dorm efficiently (monitoring) and decides to look at a map (control). Thus, ecologically relevant motivations interact with metacognitive processes during EL.

The student also navigates under constraints. There are environment constraints: physical factors constrain route options. The student cannot cut across a field enclosed by a locked gate, a factor to remember for future travel decisions. The student operates under time constraints (return to the dorm before dinner or reach a destination before nightfall). Time pressure during EL can influence the cues people attend to (Credé et al., 2019) and their reliance on familiar routes (Brown et al., 2020; Brunyé et al., 2017, 2019). Thus, time constraints during navigation can influence cues that are processed and navigation decisions. When running late, we might take a shortcut if we are confident in our environment knowledge, feel it is an option, and can handle the cognitive demand required to flexibly navigate (Boone et al., 2019; Brunyé et al., 2017)*.*

Finally, the student’s behavior suggests cognitive constraints are at play. Specifically, environment complexity increases navigation uncertainty (Slone et al., 2016). Uncertainty may then lead one to review a map to help find their way, thus engaging in metacognitive control. Uncertainty can also impose additional cognitive constraints. Uncertainty increases cognitive load via working memory requirements (Coutinho et al., 2015). A higher cognitive load leaves fewer cognitive resources for other tasks, such as encoding environment information for later use (Coutinho et al., 2015). Thus, EL appears to come with inherent cognitive constraints. Put simply, environments are information rich, and it is not possible to remember everything. In this review, we highlight the need to consider ecologically relevant motivations and constraints, including environment, time, and cognitive constraints, throughout research on metacognition and EL. These EL characteristics should subsequently shape methodological approaches, or the way metacognition can be investigated in EL contexts. Throughout this review, we describe these methodological approaches in greater detail and provide recommendations for investigating metacognition’s role during EL.

### Metacognitive monitoring and control interact to support EL

The final consideration we discuss is that metacognitive monitoring and control interact to support EL. Metacognition is commonly defined by separating it into two parts: monitoring and control (Nelson & Narens, 1990, 1994). These components have been supported by verbal learning research (Double et al., 2018). In our scenario, the student monitors the mnemonic cue that they do not remember the route back to the dorm and reviews a map (control). This example captures how monitoring and control interact to support EL. Critically, monitoring EL uncertainty would provide little benefit without the ability to exercise control, gain additional information, and successfully navigate to their destination. Here, we consider how monitoring and control interact to support EL. In doing so, we offer recommendations for how theoretical and methodological approaches can incorporate this consideration.

### Overview

To review, environments are complex. People use many different cues during EL. We observe various landmarks (local external cues) and their relative locations. We may code landmark locations relative to ourselves (internal spatial cues) or note the sun’s setting location (global environment cue) to further orient. We use mnemonic cues to decide whether to review a map thereby engaging in metacognitive control to gain more information.

**Fig. 2:**
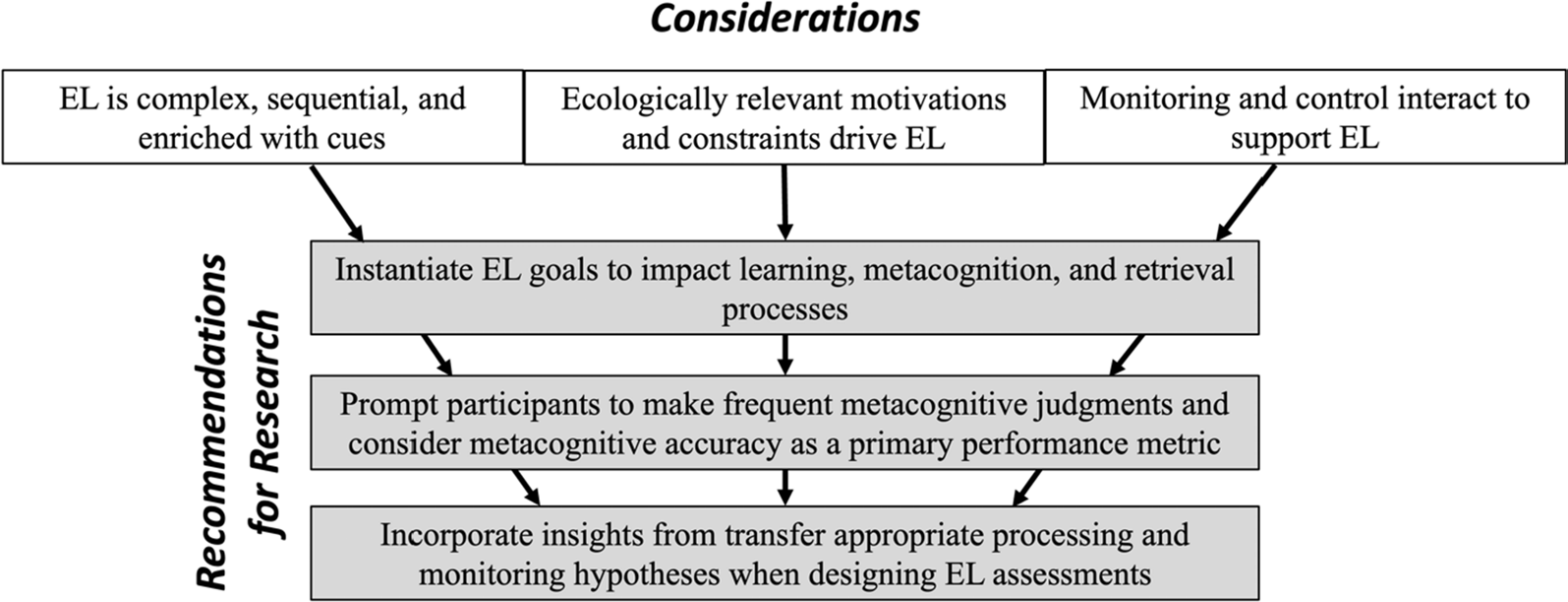
Summary of considerations and recommendations for investigating metacognition’s role in EL

We learn environments while engaged in ecologically relevant motivations (buy books, make it to dinner) and under various constraints (environment, time, cognitive). Both processing unfamiliar information and being uncertain about information increase cognitive load (Aretz, 1988; Coutinho et al., 2015; Tuovinen & Sweller, 1991). People use navigational aids when they are unfamiliar with or unsure about an environment, based on a sense of effortful processing (a mnemonic cue). Therefore, metacognitive monitoring and control processes interact to support EL (Fig. 2).

We propose that these three considerations about EL prompt recommendations for the way metacognition is investigated during EL. Specifically, researchers should: (1) instantiate EL goals to impact learning, metacognition, and retrieval processes, (2) prompt learners to make frequent metacognitive judgments and consider metacognitive accuracy as a primary performance metric, and (3) incorporate insights from transfer appropriate processing and monitoring hypotheses when designing EL assessments. Incorporating these ecological and methodological considerations into research theory and design may promote effective exploration of metacognition’s role during EL. Such research should uncover ways to bolster metacognitive gains and subsequently inform interventions to aid wayfinding success among early environment learners.

### Recommendation #1: instantiate EL goals to impact learning, metacognition, and retrieval processes

EL requires encoding visual, spatial, and verbal information (landmark names). Additionally, EL is a process that unfolds over time and space. This means aspects of the environment are learned sequentially and need to be integrated over time (Ishikawa & Montello, 2006; Siegel & White, 1975). Because of EL’s complexity, it is not possible to remember everything and learners risk attending to cues that do not reliably indicate future memory success.

Attending to unreliable or uninformative cues while learning can impede metacognitive judgment accuracy and memory success. Various circumstances impact cue reliability, and the relative weight assigned to cues may differ across conditions, time, and motivations (Koriat, 1997). For instance, processing nearby, compared to distant, landmarks (internal spatial cues) results in greater location precision when returning to a remembered destination (Sjolund et al., 2018; Zhao & Warren, 2015). A landmark’s physical properties (external cues), such as a particularly ugly purple building, can also influence cue reliability (Chen et al., 2017). However, in verbal learning, surface-level features of text do not reliably indicate verbal comprehension (Thiede et al., 2010). Thus, some of the same cues that are less predictive in verbal learning contexts could in fact be diagnostic in EL contexts. Finally, beliefs about one’s general or specific memory ability (e.g., I am bad with directions) function as mnemonic cues. When combined with other cues, mnemonic cues may also be more or less diagnostic of future memory (Koriat, 1997). In sum, some cues are more reliable than others and this reliability can vary for many reasons.

Cue reliability can also be driven by instruction. Telling participants a cue is reliable can influence performance, independent of actual cue reliability (Jaeger et al., 2012; Selmeczy & Dobbins, 2017). Thus, it is possible to guide learners to utilize certain cues over others in pursuit of a goal. One way to guide attention and bolster learning is by instantiating goals. Learning goals impact memory and attention (Anderson & Pichert, 1978; Kelly & McNamara, 2010; Pichert & Anderson, 1977; Taylor et al., 1999) and interact with metacognitive processes (Mitchum et al., 2016). Therefore, we recommend that investigators instantiate learning goals to guide attention to goal-relevant cues and subsequently impact learning, metacognition, and retrieval processes.

#### Learning goals: encoding and retrieval

Environments are complex and difficult to learn. Additionally, EL goals can vary. For instance, one student may need to learn how to navigate from their dorm to the library and back, another student may just need to look at a map to learn the name of the nearby coffee shop, and a third may need to know the overall campus layout to deliver campus mail. There is a relationship between EL goals and the information encoded (Taylor et al., 1999). We suggest that learning goals may benefit encoding and memory outcomes and provide more ecologically valid data for researchers.

Learning goals influence knowledge gained about an environment by increasing attention to goal-relevant information (Brunyé & Taylor, 2009; Taylor et al., 1999). Participants were told to learn the environment layout (survey goal) or the fastest way between locations (route goal). Spatial memory was assessed through survey-based and route-based tasks. People who learned with a survey goal were more accurate at survey-based tasks, while those with a route goal were more accurate at route-based tasks. These findings suggest that learning goals guide the encoding process and promote better memory for certain aspects of an environment (Taylor & Naylor, 2002). This means researchers can guide encoding processes by providing environment learners with specific learning goals. With goals, learners direct attention to goal-aligned information. To this end, goal-relevant information is more likely learned and later recalled (Pichert & Anderson, 1977).

Goals can also guide memory retrieval. Anderson and Pichert (1978) found that instructing subjects to recall a story for a second time from a different perspective resulted in production of additional information related to the new retrieval perspective. In other words, when the elements deemed important changed, subjects remembered previously unrecalled information in alignment with their new recall goal (Anderson & Pichert, 1978). These findings suggest that goals are fundamental to both encoding and retrieval. Specifically, providing learners with goals can help guide attention to important components during learning and at test. In sum, researchers can instantiate EL goals not only to impact cognitive processes at encoding, but also during retrieval.

#### Learning goals: metacognition

Learning goals also share a relationship with metacognitive processes (Thomas et al., 2022). We described how learning goals can guide attention to goal-relevant cues and aid memory through encoding or retrieval processes. Metacognitive monitoring prompts can similarly impact encoding. Rating one’s likelihood of remembering information in the future can promote retention of learned materials, potentially by influencing the way people attend to information (Janes et al., 2018). In other words, metacognitive monitoring processes can reinstate attention for goal-relevant information.

In addition to attending to goal-relevant *spatial* cues, learners must manage the influence of *mnemonic cues*, or their subjective cognitive and metacognitive processes throughout EL. Spatial research finds that subjective confidence in wayfinding ability can impact subsequent strategy use (Picucci et al., 2011). Learners must integrate information from both spatial and mnemonic cues to achieve EL goals. In our scenario, the student must integrate their environment knowledge with their mnemonic sense of whether this knowledge is sufficient to navigate back to the dorm. Monitoring these combined cues informs their decision to refer to a map (metacognitive control).

Little is known about the cues learners use to predict future memory success in EL. Learners can refer to multiple goal-related cues to formulate their predictions. However, they can also refer to goal-unrelated cues, which may be less predictive of actual memory. In fact, people integrate multiple cues during metacognitive judgment formation (Koriat, 1997; Undorf et al., 2018). In verbal learning research, even cues such as font size impact predictions of future retrievability. Subjects rated words in a larger font as more recallable than those in smaller font (Rhodes & Castel, 2008). Actual recall did not vary based on font size. This result emphasizes the notion that metacognitive judgment formation is sometimes subject to nondiagnostic cues, even for relatively simple materials such as word pairs or written passages.

Early evidence on the nature of metacognitive judgment formation while learning from maps provides intriguing findings on cue susceptibility (Stevens & Carlson, 2019). Participants studied a low-detail or high-detail map. After study, they made a series of JOLs to rate their confidence on how well they learned each building and the map as a whole. They also rated their confidence in being able to point to environment landmarks. At test, the deviation between the participant’s imagined viewpoint and the map north was manipulated. In line with verbal learning research, participant judgments were sensitive to perceptual aspects of the stimuli. Namely, participants provided higher JOLs with detailed maps. However, this study’s findings have an added level of complexity. Only learners in the high-detail condition could predict their later performance (JOLs), suggesting that perceptual detail was relevant in predicting future performance. The relevance of perceptual detail is contrary to verbal learning findings, where stimuli attributes (e.g., font size) result in higher but inaccurate JOLs (Rhodes & Castel, 2008). The researchers conclude that high-detail maps may facilitate performance when learners need to flexibly adopt perspectives in an environment, and this facilitation is subjectively reflected by the learner’s confidence judgments (Stevens & Carlson, 2019).

Interestingly, how perceptual detail impacts metacognitive monitoring accuracy in EL may depend on the demands of the task. Research on geospatial display design found that metacognitive judgments from novice and skilled learners indicated a preference for geographically complex displays (Hegarty et al., 2012). Despite this preference, more complex, realistic displays produced slower, less accurate comprehension as compared to simpler maps. One reason this may have been the case is more complex displays included extraneous or irrelevant information. These results offer an early clue into how findings from metamemory research with verbal materials may require reconsideration in EL contexts.

Additionally, achievement goal orientation can impact metacognitive judgments during learning, even when actual performance is not impacted (Ikeda et al., 2016; Kroll & Ford, 1992; Zhou, 2013). Some may wonder why accurate metacognitive judgment formation is so critical if actual performance is not impacted. In EL, learners need to accurately gauge whether they will remember the environment in the future, else they may get lost. Effective metacognition promotes better utilization of time resources, such as only allocating study time to environment elements one is unlikely to remember (DeCaro & Thomas, 2019; Dunlosky & Connor, 1997; Tullis & Benjamin, 2012). Accurate metacognitive monitoring can also optimize our understanding of which environment cues are diagnostic of the current learning state (Koriat et al., 2006; Nelson & Leonesio, 1988). To promote effective translation of experimental findings in laboratory settings to more applied contexts, research on metacognition and EL needs to target understanding of learning efficiency.

In metacognitive research with verbal learning materials, learners can effectively modify their learning goals to promote learning efficiency. The act of making JOLs can influence study goals by drawing attention to the idea that some materials will or will not be remembered (Mitchum et al., 2016). Therefore, learners who make JOLs may de-emphasize a mastery goal, such as to remember all word pair associates. Instead, when these learners view several word pairs, they can selectively shift learning to easier-to-remember pairs and neglect more difficult pairs. This has been understood as the *changed-goal hypothesis.*

These findings provide insight on the reactive role of JOLs during learning and suggest that prompting learners to monitor the learning process influences subsequent control and learning outcomes. However, such hypotheses cannot necessarily be directly translated to navigation contexts due to fundamental differences between environment and word pair learning. Taking our scenario where a student travels from their dorm to the bookstore, it is not feasible to only attend to the easier segments in their route while de-emphasizing more difficult ones (route segments with several decision points). Segments of routes necessarily combine, whereas word pairs do not.

#### Summary

Future research on metacognition’s role during EL is required to clarify the applicability of existing metacognition hypotheses in this complex visuospatial setting. Ultimately, goals impact metacognitive processes such as monitoring, which in turn influences learning strategy regulation, or metacognitive control (Kroll & Ford, 1992; Zhou, 2013). In EL, inaccurate metacognitive monitoring can result in misdirected self-regulation, ineffective learning, and real consequences for navigators. Investigators need to understand the relationship between learning goals, metacognition, and retrieval when developing EL research. We suggest that learning goals impact encoding, metacognition, and retrieval processes in EL. Giving learners explicit learning goals can guide attention during encoding and information access at retrieval. Additionally, motivations and subjective states impact the cues used in metacognitive judgments, and in turn, guide self-regulation to promote or disadvantage retrieval success.

Methodologically, leveraging goals to guide learning, metacognition, and retrieval benefits memory and theoretical outcomes. Narrowed goal instantiation provides researchers with a more focused assessment of the cognitive processes at play during EL. If instead learners are simply told to learn an environment and are given an opportunity to navigate before memory is tested, researchers cannot identify relevant cues and cognitive processes driving metacognitive accuracy and long-term learning. This is because the cues people can refer to during EL are inherently vast. Free exploration of an environment may result in poor learning, metacognitive accuracy, and later memory (Kintsch, 1998; Kirschner et al., 2006; Snow & Lohman, 1984). Additionally, a learner may have good memory for some aspects of the environment, but the test does not evaluate this knowledge. In other words, there can be a mismatch between what learners attend to at learning and what is tested (Blaxton, 1989; McDaniel et al., 1978; Morris et al., 1977; Nairne & Widner, 1987; Rodrigues et al., 2019; Thomas & McDaniel, 2007). A free-exploration approach also limits our ability to provide practical recommendations to improve learning, predictive accuracy, and memory. More importantly, environment exploration free from goals rarely occurs. Therefore, EL research may benefit from specifying learning goals.

### Recommendation #2: prompt learners to make frequent metacognitive judgments and consider metacognitive accuracy as a primary performance metric

We explained how environment learners have many available cues to monitor learning, inform predictions of future memory success, and determine whether they metacognitively control learning. Furthermore, because learners cannot remember every environment detail, they must prioritize certain cues. This is a tricky characteristic about EL; not all cues reliably indicate environment memory or successful future wayfinding. Cues influence learning and judgments of future retrievability. Therefore, it is important for learners to frequently monitor their likelihood to remember environment components.

In addition to their importance in helping us achieve our wayfinding goals, the cues we attend to during EL can impact the relationship between metacognitive monitoring and retrieval success. Accurate monitoring can lead someone to metacognitively control learning in a beneficial way (Nelson & Dunlosky, 1991). For instance, a learner may decide to restudy difficult to retrieve information and thereby promote future retrieval and wayfinding success. These points suggest that accurate monitoring should be a primary measure of EL success. Studying prediction accuracy allows researchers to assess cue diagnosticity. Therefore, we suggest that memory accuracy should be weighed alongside metacognitive accuracy (positive relationship between prediction and performance) to effectively gauge EL success. To provide a comprehensive assessment of metacognitive accuracy, we recommend that learners make frequent metacognitive judgments.

There are many cues that people can attend to during EL, and these cues change as one navigates from one location to the next. Even during map learning, people scan the entirety of the map and shift their attention to different elements over time (Brunyé & Taylor, 2009). Practically, metacognitive monitoring judgments can be valuable because they can provide insight into the learner’s cognitive processes. For instance, if a learner rates themself as unlikely to remember certain routes over others, assessing those route components can offer clues as to what cues learners use to form monitoring judgments. Findings from multimedia research also weigh the importance of metacognition during complex learning; however, metacognitive skills are typically measured separately from the learning task and correlated with test outcomes (Cuevas et al., 2002, 2004; Ford et al., 1998; Schwartz et al., 2004; Scott & Schwartz, 2007). This approach offers little insight on the learner’s cognitive processes as they interact with learning stimuli. Such insight may be particularly useful for research with complex spatial materials.

Metacognitive monitoring judgments can also influence the way learners attend to or encode information, also known as judgment reactivity (Double et al., 2018; Janes et al., 2018). The reactive benefit of JOL’s is primarily demonstrated in verbal learning research. The idea that prompting learners to engage in metacognitive monitoring processes can benefit learning outcomes also aligns with the earlier discussion on leveraging goals to impact encoding, retrieval, and metacognition. In other words, encouraging metacognitive monitoring through structured judgment prompts can positively impact environment memory. Given prior observations on the positive, reactive role of making metacognitive judgments for verbal materials (Janes et al., 2018; Soderstrom et al., 2015), we propose that similarly positive impacts may be observed in EL contexts. Namely, some aspects of EL are verbal (landmark names or navigation instructions). Additionally, recent research from Mason et al. (2022) finds that the act of rating one’s likelihood to remember landmark name pairs with and without the context of a map results in better memory across both contexts compared to not making such judgments. Therefore, there is reason to believe that the act of monitoring EL can be beneficial to learning.

Insights into the environment learner’s cognitive process and the potential for judgment reactivity both benefit from participants making frequent metacognitive judgments. Doing so increases measurement sensitivity. For example, having learners make JOLs for certain routes provides by-route information on the learning experience and promotes frequent opportunities to engage in metacognitive control. If someone rates themself as unlikely to remember a route, they may want to restudy that route before navigating, and/or they may want to attend to different environment cues to gain more helpful information moving forward. This example also emphasizes why metacognitive monitoring accuracy is an important primary performance metric. Faulty monitoring can lend itself to poor self-regulation and impair learning.

In contrast, prompting only global or infrequent metacognitive judgments, a strategy used throughout metacomprehension research (Dunlosky et al., 2005), has two important limitations. Namely, researchers gain indistinct information about the learner’s cognitive processes, and opportunities for JOL-reactivity are limited. Global monitoring judgments only provide a broad understanding of the learner’s subjective experience. Specifically, it would remain relatively unclear which cues learners use to formulate metacognitive monitoring during EL. Prompting frequent judgments can provide insights into which environment information results in low versus high ratings. As an example, routes involving a relatively high number of turns may be rated differently than those with few turns. This example can also be translated to map viewing, where map complexity may impact judgment formation. Taking these factors into account, it is necessary for judgments to target a finer grain size, otherwise described as “term specific” judgments (Dunlosky & Lipko, 2007; Dunlosky et al., 2005). While verbal learning research also embraces “term specific” judgment methodologies, the additional complexity of EL materials, with verbal (landmark names), visual, and spatial properties, may make a by-item judgment approach more necessary. As an example, having learners make judgments after each route segment, or as they approach intersections where a decision is required (Brunyé et al., 2018), may yield important insights into environment cue use. To this end, “term specific” judgments in verbal learning research may be redefined as “turn specific” judgments in EL methodologies. This finer grain size translates to more specific takeaways that can benefit future iterations of metacognition and EL experimentation.

Next, infrequent metacognitive judgments limit metacognitive monitoring and self-regulation engagement. Earlier, we considered how metacognitive monitoring and control interact to support EL. The act of making JOLs may benefit memory (e.g., reactivity). JOL-reactivity has been shown repeatedly with word pair stimuli (Dougherty et al., 2005; Soderstrom et al., 2015; Zechmeister & Shaughnessy, 1980), which we suggest are less complex than environments. Environments also contain word pairs (landmark name pairs). But, these landmarks also have geographic locations that can be conceptualized relative to one another and to other environment features. We may learn to navigate from the dorm to the psychology building (point *a* to point *b*) and from the psychology building to the bookstore (point *b* to point *c*), but later need to extract knowledge to navigate from the dorm to the bookstore (point *a* to point *c*). In other words, while EL includes learning names, like word pair learning, it also includes visually and spatially complex properties. Furthermore, environment “pairs,” (such as multiple landmarks), are often used in different combinations, requiring a different cognitive approach compared to word pair memory tests where the pairs are fixed.

Environment complexity further supports the need for learners to frequently engage in metacognitive monitoring. Because EL occurs over time and space and there are a variety of cues at play, learners must frequently update the cues they attend to. In a somewhat simplistic scenario where a learner only attends to landmark cues (this could entail attending to landmark locations, names, and/or other physical features), they would still have to update which landmarks they attend to as they move along a route. Because JOL-reactivity benefits likely influence the way people attend to or encode information, it is necessary to have learners make frequent judgments. Some verbal learning designs similarly prompt participants to provide judgments for each word pair.

#### Summary

In sum, learners should make frequent metacognitive judgments to provide finetuned insights on the learner’s cognitive processes and to promote JOL-reactivity. Next, metacognitive accuracy, or the relationship between monitoring judgments of future retrievability and actual retrieval success, should be a primary performance metric. Memory success is necessary for people to successfully wayfind.

When someone is actively learning an environment, the ability to accurately gauge their learning success is critical. As discussed earlier, accurate monitoring lends itself to self-regulation strategies that benefit learning and later memory (Dunlosky & Lipko, 2007). A wayfinder who accurately deems themself unlikely to remember may be more successful than one who inaccurately deems themself likely to remember only to become disoriented or lost. In other words, inaccurate EL monitoring judgments carry important consequences. Therefore, environment learners must introspect and correctly gauge the signals that external, internal, and mnemonic cues provide to formulate accurate predictions of future memory success. Findings from the literature on transfer appropriate processing (TAP) and transfer appropriate monitoring (TAM) provide insights on what factors can impact memory and monitoring accuracy among learners.

### Recommendation #3: incorporate insights from transfer appropriate processing and monitoring hypotheses when designing EL assessments

Our final suggestion is to incorporate insights from transfer appropriate processing (TAP) and transfer appropriate monitoring (TAM) hypotheses when designing EL assessments. Based on the TAP hypothesis, the value of acquisition activities should not only be defined relative to learning goals, but also to tests that are congruent with these goals (Blaxton, 1989; McDaniel et al., 1978; Morris et al., 1977; Nairne & Widner, 1987; Thomas & McDaniel, 2007). For instance, Morris et al. (1977) found that when they directed participants to attend to rhymes during learning, they performed better on rhyming tests than when they directed them to process semantic meanings. Classic TAP findings are also observed with materials other than word pairs. In one study, motoric processing while encoding digit strings resulted in better recognition when motoric processing was reinstated at test (Fendrich, 1998). Findings from VR research suggest that learners can effectively transfer knowledge gained from VR to real-world environments, but they perform best when tests match learning procedures (Rodrigues et al., 2019). Specifically, learners performed better on the retrieval task that involved a replication of learned routes as compared to sketch mapping or picture ordering tasks. Thus, TAP suggests directing participant attention to specific environment cues will result in better memory if later tests call upon those cues (Taylor et al., 1999). In EL contexts, strategically directing learners to attend to external environment cues and internal spatial cues needed to re-navigate should lead to more successful renavigation performance. Learners may similarly be guided to monitor their own wayfinding uncertainty (mnemonic cue) and subsequently refer to navigational aids (control) only as needed. Using navigational aids consistently impairs environment memory (Gardony et al., 2013, 2015). This approach may further scaffold EL and subsequent memory processes.

In addition to aiding memory, it is essential to scaffold metacognitive monitoring success, or a positive relationship between predicted and observed memory. Based on the TAM hypothesis, judgment accuracy is a direct function of the match between properties of the judgment and test (Dunlosky et al., 2005). However, this hypothesis is also largely tested with verbal materials. Weaver and Kelemen (2003) acknowledge that more complex materials allow for a wider range of encoding strategies and processing during judgments and test; therefore, it might be important to match processing for such materials. However, the more complex stimuli referred to by these researchers are text passages, which are arguably less complex than environments or map displays that contain verbal, visual, and spatial information. Though TAM findings have not been tested in EL contexts, they have the potential to provide insights on how researchers can methodologically scaffold accurate metacognitive monitoring during EL.

To our knowledge, we are the first to investigate the conditions necessary for accurate metacognitive monitoring predictions during map-based EL (Mason et al., 2022). Participants were instructed to learn routes or relative landmark locations from maps. During learning, they either made judgments of learning (JOLs) about their likelihood to remember routes or relative landmark locations on a future memory test. The alignment between the learning goal and JOL prompt was manipulated such that some learners were in a condition with congruent goals-JOL prompts, while others had incongruent ones. Tests evaluated information consistent with their learning goal. Most learners were relatively accurate at predicting their future memory for the map information, except for those instructed to learn routes but monitor their likelihood to remember relative landmark locations. This study provides early insights into the complexities of metacognitive monitoring during EL from maps and suggests that the relationship between learning goals and JOL prompts may guide monitoring accuracy.

As with TAP, predictive accuracy is also enhanced when learners engage in encoding processes that match later retrieval processing (Thomas & McDaniel, 2007). Namely, controlled strategy selection is driven by metacomprehension processes. When learners do not accurately predict their future memory, it may signal misdirected attention and control efforts and likely means learners cannot efficiently regulate the encoding process or promote memory at test (Nelson & Leonesio, 1988; Thiede, 1999; Thiede et al., 2003, 2011). In other words, accurate monitoring is essential for advantageous control, successful memory, and efficient learning.

#### Summary

We suggest that environment learning researchers should consider TAP and TAM findings when designing EL assessments. For instance, guiding learners to attend to landmark names alone likely does not prepare learners to re-navigate the environment. In other words, tests of learning should be appropriately designed relative to learning goals. With that said, ecologically relevant motivations and constraints must be considered during EL experimentation. In real-world navigation, we often transfer knowledge of the route from point *a* to point *b* and from point *b* to point *c* to navigate from point *a* to point *c*. Such transfer has ecological relevance in EL research (Chrastil & Warren, 2013, 2015; He et al., 2019). In these scenarios, navigators may be required to flexibly switch the cues they attend to. For instance, if navigating from point *a* to point *c* for the first time, local cues (landmarks) may be less useful, whereas global cues (sun as an indicator of cardinal directions) may better aid wayfinding. There are multiple cues that learners can attend to and encode or fail to encode during EL (Chen et al., 2017; McNamara & Chen, 2022). These cues can inform monitoring predictions and impact self-regulation for better or for worse. Given the complex nature of these learning materials, questions about why people’s metacognitive predictions are not accurate are also complex. Because environments are generally more complex than verbal or multimedia materials, existing theoretical explanations must be adapted to account for the ways that metacognition can scaffold EL.

## Implications for the future of EL

In this age of technology, one might wonder why it is still important for navigators to engage in effective EL strategies. While technological advancements offer navigators ease, they are not always successful. Device batteries can die, and online maps may not reflect live updates on roadblocks caused by emergencies or natural disasters. In other words, the luxury of following point-by-point directions to a destination does not mean that EL skills are disposable. In fact, the contrary is true. First responders need to learn environments quickly and flexibly, with or without access to a map on a device. Given the possibility for problems during real-world, high-stakes navigation, knowing how metacognition might impact learning could play a key role in identifying methods to help environment learners. This information could strategically inform improvements to navigation app design. Research suggests that navigational aids impede memory for environments (Gardony et al., 2013, 2015). Future generation navigational aids may be designed to respond only when people are showing signs of uncertainty about the environment (Keller et al., 2020). By implementing findings from research on metacognition and EL, it may be possible to optimize learning outcomes alongside the utilization of aids.

## Conclusion

Navigators monitor environment information, form judgments about their likelihood to remember their way, and engage in control by utilizing tools to prevent getting lost. This strategic monitoring-control relationship may scaffold learning and memory. However, historical metacognitive theories and methodologies must be reconsidered in EL contexts. In this review, we clarified why standard metacognitive approaches need modifications to benefit cognitive processes during EL. We began with three considerations to weigh when exploring metacognition in EL contexts. EL is enriched with multiple different types of cues and driven by ecologically relevant motivations and constraints. This complicates our understanding of the relationship between metacognitive monitoring, control, and ultimate learning outcomes. Therefore, we provided three recommendations for research to advance our understanding of metacognition’s role during EL.

Our recommendations aim to methodologically support the interactive metacognitive process and concurrently gain more informative data. Learners’ attention can be guided to informative cues through goal instantiation and metacognitive monitoring prompts. These approaches should promote better memory and metacognitive accuracy. We suggest learners should engage in frequent metacognitive monitoring. Frequent judgments provide detailed insight on learners’ cognitive processes and may encourage strategic learning regulation while bolstering metacognitive accuracy. Metacognitive accuracy should be considered a primary performance metric due to the ecological implications learners face if they incorrectly gauge their ability to remember an environment. Based on TAP and TAM hypotheses, if learners are guided to utilize predictive cues to inform their metacognitive judgments, and if tests necessarily call upon those same predictive cues, then learners should be more accurate at predicting future memory success.

A comprehensive theoretical model that accounts for task demands, learner motivations, cognitive resources, and prior knowledge is needed. Such a model may account for metacognitive processes across a wide range of tasks including those associated with EL. However, how these factors interact and contribute to the metacognitive process for complex visuospatial EL tasks remains understudied. While there are several theoretical models that account for the relationship between monitoring and control within the verbal learning domain, it remains unclear how those models would account for that same relationship in EL. We propose that task demands and learning motivations are likely strong contributors to how people engage in metacognitive processes in this context. To effectively promote metacognitive gains during EL and subsequently inform future navigation applications, researchers should incorporate both ecological and methodological considerations into theory and design. Only after we better understand metacognition’s role in EL can we apply it to improving navigation success.

## Data Availability

Not applicable.

## Abbreviations

EL: Environment learning
VR: Virtual reality
TAP: Transfer appropriate processing
TAM: Transfer appropriate monitoring
